# International Medical Congress

**Published:** 1876-07

**Authors:** 


### Miscellaneous.

### International Medical Congress.

The International Medical Congress will be formally opened at noon on Monday the fourth day of September. The sessions of the congress and of its sections will be held in the University of Pennsylvania, Locust and Thirty-fourth streets. The general meetings will be held daily, from ten to one o'clock. The sections will meet at two o'clock. Luncheon for Members of the Congress will be served daily in the University building from one to two o'clock.

On Wednesday evening, September 6th, Dr. J. J. Woodward, U. S. A., will address the Congress on the Scientific Work of the Surgeon-General's Bureau.

The public dinner of the Congress will be given on Thursday evening, September 7th, at seven o'clock.

The registration book will be open daily from Thursday, August 31st, to Saturday, September 2d, inclusive, from twelve to three P. M., in the hall of the College of Physicians, northeast corner of

Thirteenth and Locust streets, and at the University of Pennsylvania on Monday, September 4th, from nine to twelve M., and daily thereafter from nine to ten A. M. Credentials must in every case be presented.

Letters addressed to the Members of the Congress, to the care of the College of Physicians, northeast corner of Locust and Thirteenth streets, Philadelphia, during the week of meeting will be delivered at the University of Pennsylvania.

The secretaries of State and Territorial medical societies are requested to forward without delay, to the chairman of the Committee on Credentials, I. Minis Hays, M. D., 1607 Locust Street Phiadelphia, lists of their duly accredited delegates to the Congress. Delegates and visitors intending to attend the Congress are earnestly requested individually to notify immediately the same committee. This information is desired to facilitate registration, and to insure proper accommodation for the Congress.

Members intending to participate in the public (subscription) dinner of the Congress will please notify the secretary of the Committee on Entertainment, J. Ewing Mears, M. D., 1429 Walnut Street, Philadelphia.

Gentlemen intending to make communications upon scientific subjects, or to participate in any of the debates, will please notify the commission before the 15th of August.

